# Evaluations of dual attractant toxic sugar baits for surveillance and control of *Aedes aegypti* and *Aedes albopictus* in Florida

**DOI:** 10.1186/s13071-016-1937-z

**Published:** 2017-01-05

**Authors:** Jodi M. Scott-Fiorenzano, Alice P. Fulcher, Kelly E. Seeger, Sandra A. Allan, Daniel L. Kline, Philip G. Koehler, Günter C. Müller, Rui-De Xue

**Affiliations:** 1 Department of Entomology and Nematology, University of Florida, Gainesville, FL USA; 2 Anastasia Mosquito Control District, St. Augustine, FL USA; 3 Department of Chemical and Biomolecular Engineering, Johns Hopkins University, Baltimore, MD USA; 4 United States Department of Agriculture-ARS-Center for Medical, Agricultural, and Veterinary Entomology, Gainesville, FL USA; 5 United States Department of Agriculture-ARS-Mosquito and Fly Research Unit, Gainesville, FL USA; 6 Department of Microbiology and Molecular Genetics, Institute for Medical Research Israel-Canada, Kuvin Centre for the Study of Infectious and Tropical Diseases, Hebrew University, Jerusalem, Israel

**Keywords:** Oral insecticide, Mosquito lure, Sugar baits, Dual attractant bait, Olfaction

## Abstract

**Background:**

Dual attractant toxic sugar baits (D-ATSB) containing two host kairomones, L-lactic (LA) and 1-octen-3-ol (O), and fruit-based attractants were evaluated through olfactory, consumption and mortality, and semi-field experiments to determine if host kairomones could first, enhance attraction of a fruit-based (attractant) toxic sugar bait (ATSB), and second, increase the efficacy of a fruit based attractive toxic sugar bait (ATSB).

**Methods:**

Four combinations of LA and O were incorporated into the ATSB and evaluated in an olfactometer to determine if these combinations could enhance attraction of *Aedes aegypti* (L.) to the bait. *Ae. albopictus* (Skuse) and *Ae. aegypti* were used to determine bait consumption through excrement droplet counts and percent mortality, of the most attractive D-ATSB (1% LA and 1% O) from the olfactory study. Semi-field evaluations were conducted in screened portable field cages to determine if the D-ATSB applied to non-flowering plants controlled more mosquitoes than the fruit-based ATSB, and ASB. Mosquitoes were exposed to D-ATSB and the two controls for 48 h and collected with BGS traps. The catch rates of the BGS traps were compared to determine efficacy of the D-ATSB.

**Results:**

During olfactometer evaluations of D-ATSB, *Ae. aegypti* mosquitoes were more attracted to 1% LA and 1% O compared to the fruit-based toxic sugar bait alone. Both species of mosquito consumed more fruit-based non-toxic bait (ASB) and ATSB than the D-ATSB. For both species, percent mortality bioassays indicated D-ATSB controlled mosquitoes, as compared to non-toxic control, but not more than the fruit based ATSB. Semi-field evaluations, BioGents sentinel traps at 48 h confirmed that ATSB (positive control) controlled *Ae. albopictus*, but there was no statistical difference between ASB (negative control) and the D-ATSB. No differences were observed between the mosquitoes caught in any of the experimental formulations for *Ae. aegypti*.

**Conclusions:**

L-lactic (1%) and 1-octen-3-ol (1%) added to a fruit-based sugar bait increased attraction of *Ae. aegypti* and may have future implications in mosquito trapping devices. The addition of the host kairomones did not enhance the consumption and efficacy of the ATSB in laboratory or semi-field evaluations for both mosquito species. We attribute to the absence of other host cues leading to lack of alighting onto bait surfaces to imbibe the toxic bait, as well as a possible decrease in palatability of the bait caused by the addition of the host kairomones.

## Background

Throughout Florida’s subtropical environment, *Aedes albopictus* (Skuse) is a commonly encountered mosquito species known to vector pathogens in urban settings. Another urban mosquito species that vector similar pathogens, *Ae. aegypti* (L.) have been steadily being reintroduced to places in Florida where they had previously been displaced by *Ae. albopictus* (Anastasia Mosquito Control District, personal communication). Florida is a major tourist hotspot, ranking number 18 out of the world’s top travel destinations [1]. Since January 2016, there have been six travel-related cases of Chikungunya (CHIKV), 40 travel related cases, with two locally acquired cases of dengue (DENV), and 832 travel related cases, with 169 locally acquired cases of Zika in Florida [2]. With a constant flow of national and international tourists, a subtropical climate, and the possible reintroduction of *Ae. aegypti*, it may be a matter of time before disease incidences of DENV, CHIKV and Zika cease to be predominantly travel-related, permanently establish in the local vector population.

More environmentally friendly adult mosquito control methods such as the ‘attract and kill’ methods of ATSBs [3, 4] have the potential to reduce mosquito populations, thus reducing probability of localized establishment of these viruses in mosquito populations. Although ATSBs have proven effective in killing mosquitoes from many genera [3, 4], in the last decade most modifications of each ATSB formulation have been changes in the fruit-based attractant or active ingredient, which only capitalize on the resting and sugar-seeking behaviors to control mosquitoes. Mosquitoes utilize a wide range of chemicals to locate resources such as, environmental sugars, mates, hosts for blood feeding, and oviposition sites [5, 6]. In order to locate these resources, insects have odor reception neurons that are highly attuned to the specific resources [7]. In many mosquito species, the female mosquitoes display a pattern of sugar and host seeking behavior that is partially mediated by odor reception [8]. Both host seeking and sugar-seeking behaviors of mosquitoes could be capitalized on by incorporating host kairomones into fruit-attractant toxic sugar baits, which could create a more “mosquito-centric”, dual attractant ATSBs, for use in mosquito control and mosquito surveillance.

Novel methodologies that incorporate fruit-based attractants can have multiple uses in mosquito surveillance and mosquito control. Foster & Hancock [9] proposed the utilization of plant-based sugars in mosquito population surveillance and control if they could attract large numbers of mosquitoes. The fruit-based attractants utilized in attractive sugar baits (ASB) can be used in conjunction with mosquito traps in mosquito surveillance to establish species and numbers of vectors in an area. Host seeking behaviors have been exploited in mosquito trapping devices, such as BioGents Sentinel (BGS) traps with the addition of BGS lure (lactic acid, ammonia, caproic acid), Mosquito Magnet™ baited with CO_2_, and Center for Disease Control (CDC) traps modified to include CO_2_ for multiple species collections in operational surveillance [10–12]. Studies utilizing human-host kairomones have identified combinations of attractive lures, which provided higher capture rates than using one type of lure [5, 6, 11, 13]. Adult *Ae. aegypti* have been responsive to lactic acid in numerous studies, which makes this chemical a possible attractant for these mosquitoes [5, 6, 11, 14]. Hoel et al. [11] indicated that L-lactic acid (LA) combined with 1-octen-3-ol (O) had a great effect on mosquito capture rates in the presence of CO_2_ for *Ae. albopictus* in Mosquito Magnet traps than they did as individual attractants.

More mosquito-specific methods are needed for mosquito control and surveillance that target important disease vectors, such as *Ae. aegypti* and *Ae. albopictus* in sub-tropical environments. Established fruit attractants, chemicals, and chemical combinations previously tested could be the key to producing more mosquito-centric dual-attractant baits, which could capitalize on both sugar and blood feeding behaviors of mosquitoes. We hypothesized that there would be differences in attraction to chemical combinations of LA and O in ATSBs, and that the inclusion of the host kairomones would capitalize on the host and sugar seeking behaviors of female mosquitoes to enhance the percent mortality and consumption of dual-ATSB (D-ATSB) as compared to the simple fruit-attractant ATSB. We addressed these hypotheses through two research objectives using mosquito attraction, excretion, and mortality as indices of preference. The first objective of this study was to determine if the integration of host kairomones, LA and O, in an experimental ATSB could increase the attractiveness of this bait creating a D-ATSB. The second objective was to determine the efficacy of D-ATSBs for use in mosquito abatement programs to control adult populations of *Ae. albopictus* and *Ae. aegypti*.

## Methods

### Preparation of ATSB solutions

Frozen mango chunks or pulp (acquired from local groceries) was sieved through a kitchen strainer (Farberware® 18-cm, Meyer Corporation, Vallejo, CA, USA) to remove fruit fibers and produce mango puree. Lime juice (1:16 v/v) (Mott’s LLP, Plano, TX, USA) was added to the mango puree as a preservative. White granulated sugar (Great Value, Walmart-Store Inc., Bentonville, AR, USA) was subsequently added at a 1:1 w/v ratio and the mixture was heated to 100 °C to make a simple syrup [15]. Reverse osmosis water was added to the mango syrup at a 3:1 v/v (water: syrup) ratio and heated slightly until sugar was dissolved, producing ASB.

Attractive toxic sugar bait was prepared from ASB by adding powdered boric acid (1% w/v) (≥ 99.5%, Sigma-Aldrich, St. Louis, MO, USA) and slightly heating the mixture for 2–5 min at 50–60 °C until the boric acid was completely dissolved.

Dual attractant toxic sugar baits were prepared by incorporating combinations of L-lactic acid (LA) (≥ 98%, Sigma-Aldrich) and 1-octen-3-ol (O) (≥ 98%, FCC, FG, Sigma-Aldrich) to ATSB solutions. ASB and a 10% sucrose solution were the negative controls while ATSB (1% boric acid) was used as a positive control. Agricultural spreader sticker, Poly Control 2 (Brewer International, Vero Beach, FL, USA) was included in the ASB, ATSB and D-ATSB formulations to increase the duration in which the baits stay on vegetation. Unless otherwise noted, all baits were dyed with 0.5% v/v green or red food coloring (McCormick & Co., Inc, Hunt Valley, MD, USA), so that the ingestion of bait could be confirmed through excrement droplet analysis and to verify that the baits evenly covered foliage surfaces during experiments.

### Olfactometer evaluations of dual-attractant combinations incorporated into ATSB

Evaluations were conducted with colonized United States Department of Agriculture (USDA) strain *Ae. aegypti* reared in accordance with Gerberg et al. [16]. Adult mosquitoes were maintained in cages in an insectary with 10% sucrose solution *ad libitum* at 80% RH and 26.7 °C under a 14:10 (L:D) photoperiod. Adult 5-7 day old female *Ae. aegypti* were utilized in these experiments. Most species of mosquito’s guts are still developing from 1–3 days after pupation, during this time they predominantly feed from sugar sources [17]. After 3 days old they begin host-seeking behaviors [16], the selection of 5–7 day old mosquitoes in these trials was to ensure that the mosquitoes displayed both sugar and host-questing behaviors.

To compare attraction of mosquitoes to different concentrations of D-ATSBs a triple-cage dual-port olfactometer was used [18]. Briefly, charcoal-filtered air that was humidified and warmed (27 + 1 °C; 60 ± 4% RH) flowed through the olfactometer with air moving at 28 ± 1 cm/s flowing through each port. Each cage consisted of two ports, which could contain a treatment or control. Each port also contained a screen cage to contain mosquitoes that flew upwind to the treatment or control. Those mosquitoes contained by the screen were counted as attracted. For each trial, approximately 60 female mosquitoes were collected from the holding cage using a draw box [19] with a human hand as attractant, thus, the preselected mosquitoes were known to be active and exhibited host-seeking behavior. The mosquitoes were then placed into each test chamber of the olfactometer. Once loaded into the olfactometer test chamber, the mosquitoes were allowed to acclimate for 40 min. Negative controls consisted of empty test ports. To verify that mosquitoes were responsive during each 20 min test period, a positive control (human hand) was evaluated. The mosquitoes attracted to the hand in the collecting tube served as a preset baseline value for host seeking response during the trials. If mosquitoes were not reactive to the preset value, tests were not conducted. All materials and olfactometer components were handled with gloves to avoid contamination with skin compounds and the olfactometer was washed with soap and water between each trial.

Trials consisted of testing ASB, ATSB, and four dual-attractant combinations (0.01% LA and 0.01% O, 0.01% LA and 1% O, 0.1% LA and 1% O, and 1% LA and 1% O). A treatment solution (3 ml) was placed in a disposable plastic Petri dish onto a small shelf in the olfactometer port. The second port was left blank as a control. Numbers of mosquitoes in both ports were counted at the end of 20 min and the number of mosquitoes remaining in the olfactometer cage was recorded and mosquitoes were removed from the olfactometer. Data from each replication represented the percentage of mosquitoes that responded to the formulation at the end of the 20 min trial time. Data was averaged over 15 replications for each formulation, and utilized for statistical analysis of responses to the formulations.

### Consumption of 1% LA and 1% O D-ATSB using excrement droplet counts and percent mortality

Evaluations were conducted with colonized United States Department of Agriculture (USDA) strain *Ae. albopictus* and *Ae. aegypti* reared in accordance with Gerberg et al. [17]. Adult mosquitoes were maintained in cages in an insectary with 10% sucrose solution *ad libitum* at 80% RH and 26.7 °C under a 14:10 (L:D) photoperiod. Adult 5–7 day old female *Ae. albopictus* and *Ae. aegypti* were utilized in these experiments.

A highly attractive D-ATSB combination, 1% LA and 1% O, from olfactometer trials was dyed with 0.5% green food coloring and bait consumption determined through excrement droplet counts. Mosquito excrement has previously been used to confirm ingestion of dyed baits through counting the dyed spots of excretion [20]. The dye included in the bait allows a pinpoint to determine mosquito excretion. As the mosquito excretes the dyed bait, the bait defuses in a circular or oblong pattern from the initial droplet, with the center most dyed part counting as one excrement. The experimental treatment solution consisted of ATSB + 1% LA and 1% O. The positive and negative treatment controls consisted of ATSB and ASB, respectively.

Disposable clear plastic cups (473 ml) (Dart Container Corporation, Mason MI) were used as cages. Two 1-cm holes were made adjacent from one another; one hole 1 cm from the rim of the cup and the adjacent hole was 1 cm from the base of the cup. One micro-centrifuge tube (1.5 ml) (Thermo Fisher Scientific Inc., Waltham, MA) holding 1.5 ml of treatment was sealed with a bait moistened cotton stopper (Walmart-Store Inc., Bentonville, AR, USA) and placed into the 1-cm hole made just above the bottom the of the cage. Parafilm® around the midsection of the vial served to hold it in place. A 6-cm diameter circle of cardstock (Pacon Corporation, Appleton, WI, USA) was inserted into the bottom of the cup to collect mosquito excrement. Once the treatments and card stock were in place, the opening of the cups was covered with mesh and held in place with an elastic band. Ten female mosquitoes were gently aspirated into each of the cages in the top 1-cm hole just below the rim of the cup. This hole was plugged with a cotton ball saturated with water daily throughout the experiment. At 96 h the mortality of the mosquitoes was recorded, and the cups were placed in a freezer -20 °C for 1 h. The cardstock was then removed from cages, and excrement droplets on the cards were counted under a dissecting microscope. The replicate schedule for D-ATSB laboratory studies follows.

1. *Ae. albopictus*: 1 experimental treatment (1% LA and 1% O) and 2 controls [ASB (negative control) and ATSB (positive control)] × 5 experimental cages (cup cages) × 4 preparation of treatments (newly prepared ATSB formulations) = (*N* = 60)
2. *Ae. aegypti*: 1 experimental treatment (1% LA and 1% O) and 2 controls [ASB (negative control) and ATSB (positive control)] × 5 experimental cages (cup cages) × 3 preparation of treatments (newly prepared ATSB formulations) = (*N* = 45).

### Semi field evaluations 1% LA and 1% O D-ATSB

Semi-field evaluations to compare foliage treated D-ATSB with ATSB and ASB were conducted at the Entomology and Nematology Department of the University of Florida. Adult *Ae. albopictus* and *Ae. aegypti* mosquitoes (5–7 day-old) were provided by the USDA Center for Medical, Agricultural, and Veterinary Entomology (CMAVE) in Gainesville, FL, USA. Screened portable field cages (*Lumite®*) (2 W × 2 H × 4 L m) were fastened to a solid foundation and utilized as the testing arenas. The evaluation used three cages for each species of mosquito with one cage for each treatment for a total of six cages. Each cage contained three non-flowering Indian Hawthorn plants (*Rhaphiolepis indica* (L.) Lindl. ex Ker Gawl) and two 8 oz containers with water-saturated cotton balls as water sources. Treatment formulations consisted of ASB as a negative control, ATSB as a positive control, and 1% O and 1% LA D-ATSB as the experimental treatment. For each treatment, three plants were individually sprayed with 20 ml of each treatment solution and allowed to dry for up to one hour.

After the drying period, 500 female mosquitoes were released into the cages. BioGent™ Sentinel traps (BioGents, Regensburg, Germany) baited with BioGent™ lure (lactic acid, ammonia, caproic acid) (BioGents, Regensburg, Germany) were placed into cages 48 h after the initial release. BGS traps were collected 24 h after their placement and the mosquitoes that were caught were counted. Three preparations of treatments were conducted on a new day with each formulation evaluated in each cage once. During each experimental preparation of treatments, the plants were washed with soap and water, and allowed to dry before the fresh formulations were applied to the plants. The replicate schedule for D-ATSB semi-field studies follows.

1. *Ae. albopictus*: 1 experimental treatment (1% LA and 1% O) and 2 controls [ASB (negative control) and ATSB (positive control)] × 3 semi-field cages × 3 BGS traps (collection devices) × 3 preparations of treatments (newly prepared ATSB formulations freshly applied to plants) = (*N* = 81)
2. *Ae. aegypti*: 1 experimental treatment (1% LA and 1% O) and 2 controls [ASB (negative control) and ATSB (positive control)] × 3 semi-field cages × 3 BGS traps (collection devices) × 3 preparations of treatments (newly prepared ATSB formulations freshly applied to plants) = (*N* = 81).

### Statistical analysis

Analyses of variances (ANOVAs) was conducted on the percent attraction of *Ae. aegypti* to D-ATSB combinations, the mean percent mortality and mean excrement droplet counts per mosquito, and the mean percent reduction of mosquitoes through BGS trap collection data for both *Ae. aegypti* and *Ae. albopictus* in each experiment through JMP 11 statistical software (SAS Institute Inc., Cary, NC, USA). The percent mortality data in laboratory studies were Henderson-Tilton corrected to account for the negative controls [21].

The analysis for the attraction studies of *Ae. aegypti* at 20 min in the olfactometer was conducted recognizing the study as a block design, blocked by preparation of treatments, recognizing groups [(experimental treatments: D-ATSB dilutions (ATSB +: 0.01 LA and 0.01 O; 0.01 LA and 1 O; 0.1 LA and 1 O; 1 LA and 1 O), ATSB and ASB), and empty control ports] as the main effects and the averaged arcsine [square root (attraction)] as the dependent variables.

The analysis for the laboratory percent mortality of *Ae. albopictus* at 96 h was conducted recognizing the study as a block design, blocked by preparation of treatments, recognizing groups [experimental treatments (D-ATSB: 1 LA and 1 O + ATSB, ATSB and ASB)] as the main effects and the averaged arcsine [square root (Henderson-Tilton corrected proportion mortality)] as the dependent variables. The analysis for the laboratory excrement droplet count per mosquito of *Ae. albopictus* at 96 h was conducted recognizing the study as a block design, blocked by preparation of treatments, recognizing groups [experimental treatments (D-ATSB: 1 LA and 1 O + ATSB, ATSB and ASB)] as the main effects and the averaged square root (excrement droplet count per mosquito) as the dependent variables.

The analysis for the laboratory percent mortality of *Ae. aegypti* at 96 h was conducted recognizing the study as a block design, blocked by preparation of treatments, recognizing groups [experimental treatments (D-ATSB: 1 LA and 1 O + ATSB, ATSB and ASB)] as the main effects and the averaged arcsine [square root (Henderson-Tilton corrected proportion mortality)] as the dependent variables. The analysis for the laboratory excrement droplet count per mosquito of *Ae. aegypti* at 96 h was conducted recognizing the study as a block design, blocked by preparation of treatments, recognizing groups [experimental treatments (D-ATSB: 1 LA and 1 O + ATSB, ATSB, and ASB)] as the main effects and the averaged square root (excrement droplet count per mosquito) as the dependent variables.

The analysis for the semi-field studies of D-ATSB on *Ae. albopictus* and *Ae. aegypti* was conducted recognizing the study as a block design, blocked by preparation of treatments/rotation of cage placement, recognizing groups [experimental treatments (D-ATSB: 1 LA and 1 O + ATSB, ATSB and ASB)] as the main effects and the averaged arcsine [square root (proportion of mosquito reduction from BGS trap collections)] as the dependent variables. When significant differences were observed for (attraction, percent mortality, excrement droplets per mosquito, and percent reductions), a Tukey’s HSD, or Tukey’s Students *t*-test were performed to separate the means, accepting differences at α ≤ 0.05. For all graphs, the data are displayed with untransformed means and standard error of the means.

## Results

### Olfactometer evaluations of dual-attractant D-ATSBs

**Fig. 1:**
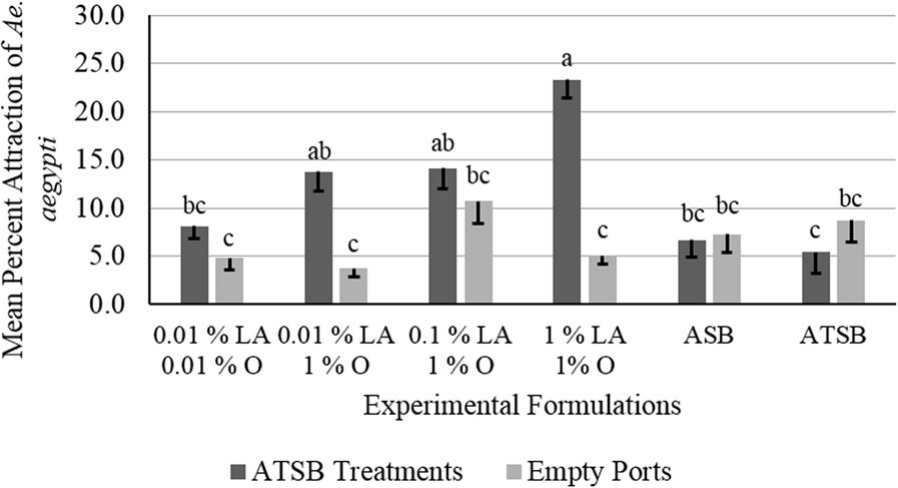
Mean percent attraction (- standard error of the mean, SEM) of adult *Aedes aegypti* to experimental combinations of ATSB and host kairomones. Differences in experimental formulations were determined at 20 min in olfactometer, between the experimental formulations and between experimental formulations and empty control ports. Differences in attraction between experimental formulations were observed between 1% LA and 1% O and the experimental formulations containing no host kairomones (ATSB and ASB). Means sharing the same letter are not significantly different at α ≤ 0.05 (Tukey’s HSD)

Significantly more *Ae. aegypti* female mosquitoes were attracted to dual-attractant combinations of 0.01% LA and 1% O and 1% LA and 1% O, as compared with the empty control ports, the other D-ATSB combinations, and the positive and negative controls (*F*_(26,154)_ = 7.14, *P* < 0.0001). These data support the hypothesis that host kairomones could enhance attraction to ATSBs (Fig. 1) and studies then expanded to examine consumption and mortality and included an additional species, *Ae. albopictus*.

### Consumption of 1% LA and 1% O D-ATSB using excrement droplet counts and percent mortality

**Fig. 2:**
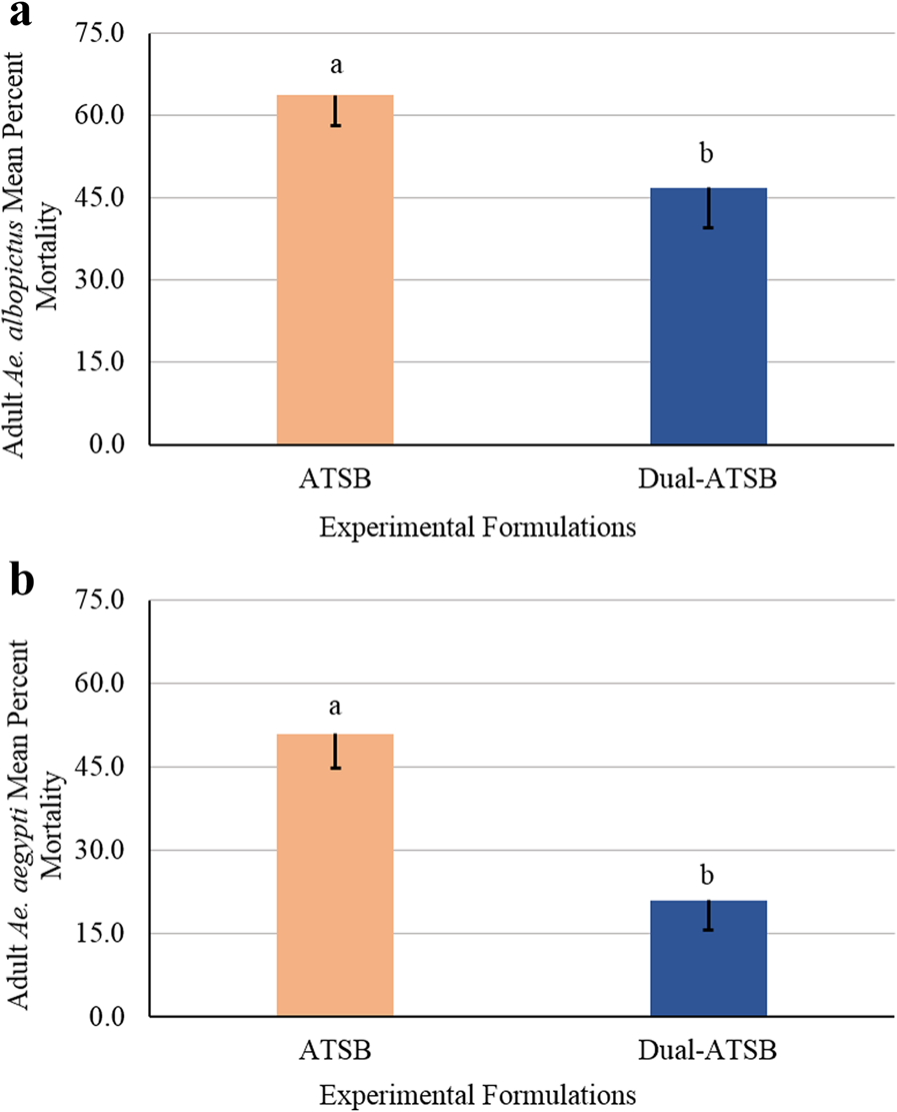
Henderson-Tilton’s corrected mean percent mortality (- standard error of the mean, SEM) of mosquitoes exposed to Dual-Attractant TSB (D-ATSB). At 96 h, the corrected percent mortality of D-ATSB was compared to the corrected percent mortality of the positive control (ATSB) to assess efficacy of bait. Means sharing the same letter are not significantly different at α ≤ 0.05 (Tukey’s HSD). **a** *Aedes albopictus* comparisons of the corrected percent mortalities of experimental formulations. **b** *Aedes aegypti* comparisons of the corrected percent mortalities of experimental formulations

**Fig. 3:**
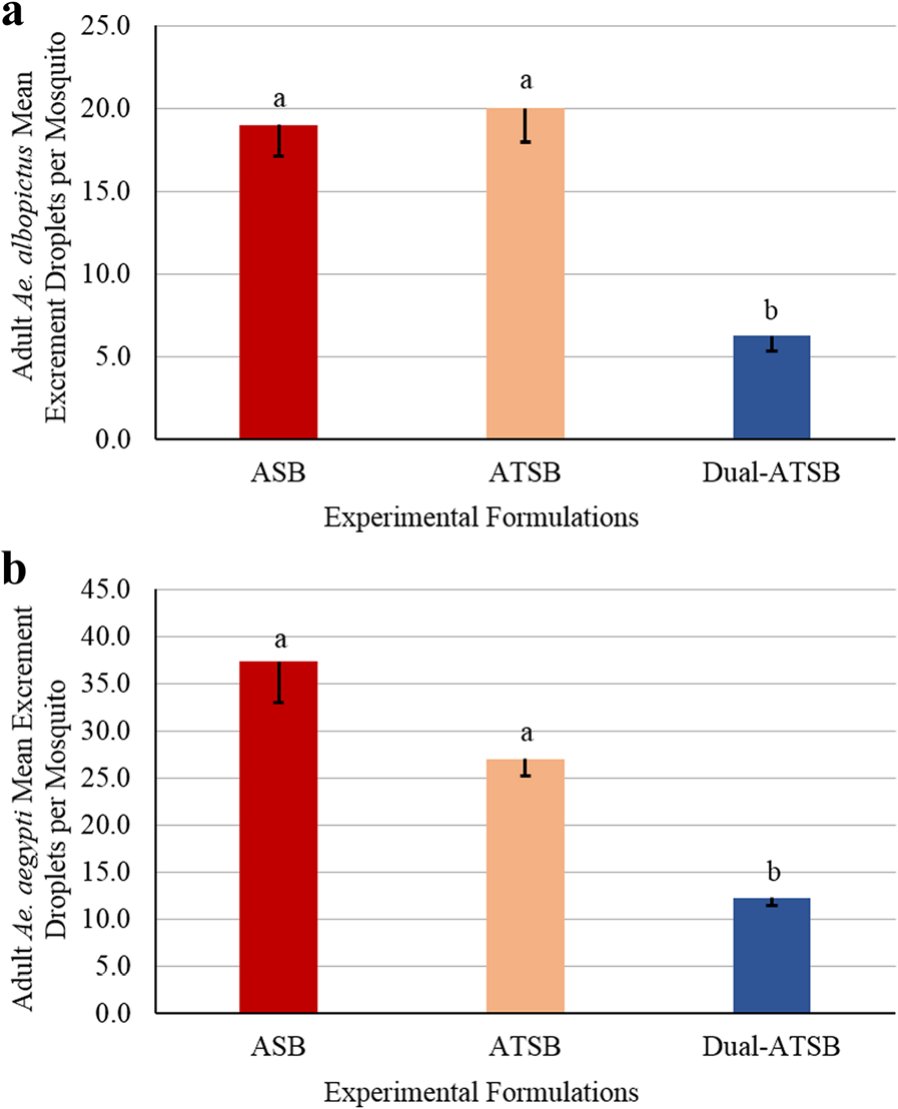
Mean excrement droplets (- standard error of the mean, SEM) per mosquito exposed D-ATSB. At 96 h, the D-ATSB excrement droplets per mosquito were compared to negative control (ASB) and positive control (ATSB) to assess excretion of bait. Means sharing the same letter are not significantly different at α ≤ 0.05 (Tukey’s HSD). **a** Excrement droplets comparisons of experimental formulations of *Aedes albopictus.* **b** Excrement droplets comparisons of experimental formulations of *Aedes aegypti*

The lack of difference in the percent mortality of both *Ae. albopictus* and *Ae. aegypti* between ATSB and D-ATSB demonstrated that addition of the secondary attractant did not enhance the percent mortality of the ATSBs (*F*_(3,32)_ = 11.26, *P* = 0.0019, and *F*_(3,26)_ = 18.92, *P* = 0.0002, respectively) (Fig. 2). Both *Ae. albopictus* and *Ae. aegypti* excreted more droplets from ASB and ATSB, than the D-ATSB (*F*_(5,54)_ = 59.55, *P* < 0.0001 and *F*_(4,40)_ = 23.46, *P* < 0.0001, respectively) (Fig. 3). These data support the null hypothesis that the D-ATSB does not enhance the percent mortality or excretion of the ATSB evaluated in this study.

### Semi field evaluations 1% LA and 1% O D-ATSB

**Fig. 4:**
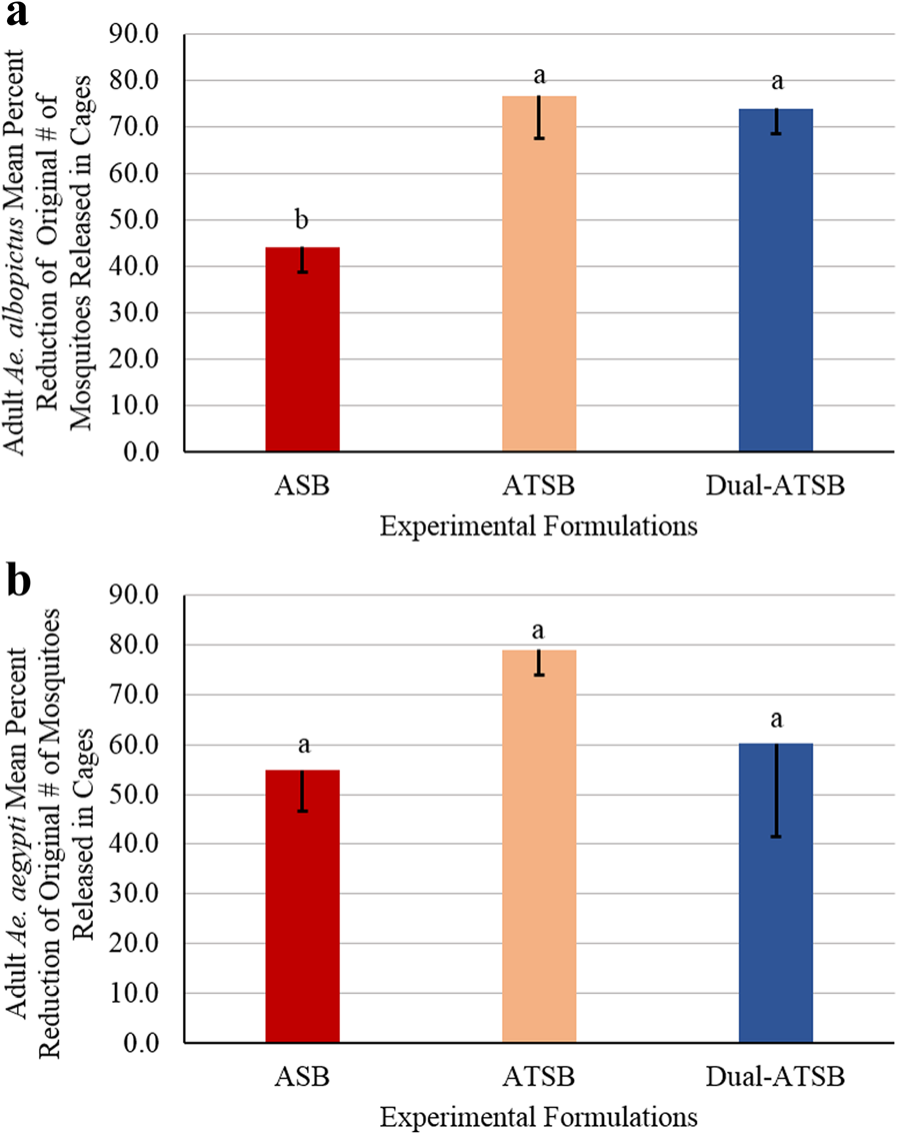
Mean percent reduction (- standard error of the mean, SEM) of adult mosquitoes exposed to D-ATSB applied to foliage. During semi-field studies, the percent reduction of D-ATSB was compared to negative control (ASB) and positive control (ATSB) to assess the efficacy of the bait. Means sharing the same letter are not significantly different at α ≤ 0.05 (Tukey’s HSD). **a** Comparisons of the percent mortality of *Aedes albopictus* exposed to experimental formulation and controls. **b** Comparisons of the percent mortality of *Aedes aegypti* exposed to experimental formulation and controls

BGS trap collections indicated that application of the D-ATSB did not lead to more control of adult *Ae. albopictus* than ATSB (*F*_(6,2)_ = 45.14, *P* = 0.0217) (Fig. 4). Similarly for adult *Ae. aegypti* evaluations, there were no differences observed in the BGS trap captures between applications of ASB, ATSB and the D-ATSB formulations (*F*_(6,2)_ = 1.41, *P* = 0.4147) (Fig. 4).

## Discussion

The olfactometer evaluations demonstrated that the inclusion of host kairomones, LA and O, in conjunction with an attractive fruit source were more attractive to *Ae. aegypti* females than just the fruit-based ATSB. Prior attraction studies have provided information on the attraction of mosquitoes to host and plant-based attractants, independently [6, 8–14, 18, 19, 22–34]. The attraction of *Ae. aegypti* to the D-ATSB combination in this study was promising both fruit and host attractants could be combined, possibly leading to improvement of mosquito surveillance techniques through incorporating these lures into active collection traps such as, BGS or CDC traps.

Current mosquito surveillance trapping only takes advantage of host kairomones to attract female host-seeking mosquitoes. Traps baited with plant or fruit-based lures are known to be capable of attracting both female and male sugar-seeking mosquitoes [28]. Foster & Hancock [9] suggested that sugar-attractants alone might be a less powerful attractant than those associated with blood. Thus, the combination of host and fruit attractants in the D-ATSB represented an enhanced potential for collection of and control of both sugar seeking male mosquitoes as well as sugar and host-seeking female mosquitoes.

Mosquito attraction demonstrated in the laboratory may not necessarily equate to consumption by mosquitoes as factors such as palatability may play a role. In the olfactometer evaluations, *Ae. aegypti* behaved in a similar fashion to that reported by Smith et al. [31] in that mosquitoes were more attracted to a dual attractant lure in the olfactometer, however that attraction did not lead to increased consumption or mortality, in either laboratory or semi-field bioassays. The percent mortality and colored bait excretion of both mosquito species in the laboratory and semi-field evaluations confirmed that the fruit-source attractant was attractive, based on scent and flavor, and therefore readily consumed by the 5–7 day-old mosquitoes. When host kairomones were incorporated in the bait, mosquitoes did not excrete the bait, and the D-ATSB failed to control more mosquitoes than the ATSB.

Insects have developed highly adaptive sensory systems of taste and smell to locate both sugar and host sources [29, 33, 35]. Sugar feeding occurs during the early onset of a female mosquito’s life and follows a cyclic pattern with blood feeding behaviors, while male mosquitoes only take sugar meals [8]. Mosquitoes rely on olfactory and possibly visual cues to locate sugar sources [9, 29]. Previous field studies have demonstrated that light colored flowers may provide possible visual cues for mosquitoes; however, since these flowers also produced a strong odor, it is uncertain if the color alone was truly a visual cue [9]. Mosquitoes utilizing visual cues for sugar location are an important topic for future studies. Volatiles in fruit that comprise their characteristic flavors and scents provide sensory cues for location of fruit [26]. Enhanced attraction response by mosquitoes to the fruit-based bait was demonstrated in this study. Our results with reduced consumption and mortality in the dual ATSB formulations suggest that the addition of the host kairomones may have rendered the bait less palatable for the mosquitoes. Further issues could have arisen due to the lack of odor plumes or additional host cues resulting in confused behavioral responses of the mosquitoes.

Mosquitoes locate hosts through chemical, heat, and visual cues [22, 23, 32, 33, 35]. Olfactory cues are generally considered long-range attractants, whereas visual cues are often short-range attractants [30]. Odor plumes, which can be short or long-range attractants, are important for olfaction detection. Mosquito antennal receptors are adapted for irregular odor plumes from hosts with the concentration and the turbulence of the odor plume affecting the mosquitos’ abilities to orient to the stimulus [33–35]. In the laboratory consumption and mortality and semi-field evaluations, the D-ATSBs were continuously offered at a discrete location (cotton ball, or on foliage). In nature, odors exist as discrete and discontinuous plumes [33]. When odors are offered to mosquitoes in a continuous fashion with no turbulence, odors may elicit anomalous behaviors [33]. In the laboratory and the semi-field trials, the consistent non-turbulent nature of the D-ATSB could have confused the ability of the mosquitoes to orient and alight on the baits, which may have caused the appearance of feeding deterrence.

Once mosquitoes detect a host through olfaction (long-range and short-range cues), they utilize “other” stimuli (short-range cues), such as moisture, heat and visual cues to guide additional behavior such as alightment on a host [32, 34]. Evaluations conducted with *Ae. aegypti* indicated that these mosquitoes will orientate toward host odor, but will fail to alight if there are not visual and heat/moisture cues associated with the olfactory cue [32–34]. During host location, tsetse flies demonstrate imprecise orientation to the odor source unless they are visually stimulated by the host or artificial target [35]. Based on the results, the absence of other stimulants may have resulted in a lack of cues guiding the mosquitoes to ingestion of D-ATSB. Further confusion could have been caused by physiological issues in resource allocation of the food once ingested.

Allocation of resources is an important physiological action of all creatures. For mosquitoes, the allocation of sugar and blood is initially diverted to different body structures with sugar resources allocated to the crop and dorsal diverticula [34, 35]. From the crop, the sugar meal is slowly utilized as energy, as needed, or stored for later usage. Blood is diverted directly to the midgut, where it is processed for usage in vitellogenesis [35]. Both resources, sugar and blood fulfill different nutritional requirements for mosquitoes, and follow different physiological pathways within the mosquito. Likewise, mosquitoes respond differently to either host or sugar sources depending on their physiological state. However, when provided both resources simultaneously, mosquitoes have physiological systems that allocate the resources according to concentration of the components. In a resource allocation study by Day [36], *Ae. aegypti* mosquitoes were fed both blood and sugar in different concentrations to determine the allocation of the resources. He confirmed results from previous studies, which stated that mosquitoes have a “switching mechanism” triggered by sugars or specific blood components (plasma, washed erythrocytes, and haemolysed blood). Once liquid is imbibed, mosquitoes are capable of distinguishing between blood and sugar concentrations and this information is used to determine the destination of the nutrients, to either the midgut or crop [36]. The D-ATSB contained host kairomones and sugar solution; no components of blood were present therefore the D-ATSB meal would have been presumably allocated to the crop and dorsal diverticulum of the mosquito.

Studies conducted by Kline et al. [12] indicated that more research is required to reveal “species” specific blends of attractants. Host kairomones included in a fruit-based toxic sugar bait attracted more *Ae. aegypti* in an olfactometer study than the fruit-based toxic sugar bait alone. Evaluation of the dual-attractant combinations indicated that consumption of the D-ATSB was negatively impacted, likely due to the presence of host kairomones. This dual formulation did not enhance the percent morality of the ATSB formulation for either species of mosquito. Future studies in the optimal concentration of host kairomones and fruit blend to optimize attraction and reduce phagodeterrency could contribute to development of this approach for localized mosquito control.

## Conclusions

This study demonstrates that L-lactic (1%) and 1-octen-3-ol (1%) added to a fruit-based sugar bait increased attraction of *Ae. aegypti* and may have future implications in mosquito trapping devices. Furthermore, this study shows that the addition of the host kairomones did not enhance the consumption and efficacy of the ATSB in laboratory or semi-field evaluations for both mosquito species.

## Abbreviations

ANOVA: Analysis of variance
ASB: Attractive sugar bait
ATSB: Attractive toxic sugar bait
BGS: BioGents sentinel trap
CDC: Center for disease control and prevention
CHIKV: Chikungunya virus
CMAVE: Center for medical, agriculture and veterinary services
D-ATSB: Dual attractant toxic sugar bait
DENV: Dengue virus
FDOH: Florida Department of Health
LA: L-lactic acid
O: 1-Octen-3-ol
USDA: United States Department of Agriculture
